# *F13A1* Gene Variant (V34L) and Residual Circulating FXIIIA Levels Predict Short- and Long-Term Mortality in Acute Myocardial Infarction after Coronary Angioplasty

**DOI:** 10.3390/ijms19092766

**Published:** 2018-09-14

**Authors:** Lucia Ansani, Jlenia Marchesini, Gabriele Pestelli, Giovanni Andrea Luisi, Giulia Scillitani, Giovanna Longo, Daniela Milani, Maria Luisa Serino, Veronica Tisato, Donato Gemmati

**Affiliations:** 1 Operative Unit of Cardiology, University Hospital S. Anna of Ferrara, 44121 Ferrara, Italy; l.ansani@ospfe.it (L.A.); jlenia.marchesini@unife.it (J.M.); gabriele.pestelli@unife.it (G.P.); giovanniandrea.luisi@unife.it (G.A.L.); giulia.scillitani@unife.it (G.S.); 2 Centre of Haemostasis & Thrombosis, Department of Biomedical and Specialty Surgical Sciences, Section of Medical Biochemistry, Molecular Biology & Genetics, University of Ferrara, 44121 Ferrara, Italy; giovanna.longo@student.unife.it; 3 Department of Morphology, Surgery and Experimental Medicine and LTTA Centre, University of Ferrara, 44121 Ferrara, Italy; daniela.milani@unife.it (D.M.); veronica.tisato@unife.it (V.T.); 4 Centre of Haemostasis & Thrombosis, Department of Medical Sciences, University of Ferrara, 44121 Ferrara, Italy; maria.luisa.serino@unife.it; 5 University Center for Studies on Gender Medicine, University of Ferrara, 44121 Ferrara, Italy

**Keywords:** myocardial infarction, myocardial healing, coagulation factor XIII, prognostic biomarkers, pharmacogenetics, left ventricular remodeling and heart failure, translation cardioprotective strategies

## Abstract

Factor XIIIA (FXIIIA) levels are independent predictors of early prognosis after acute myocardial infarction (AMI) and the Valine-to-Leucine (V34L) single nucleotide polymorphism (SNP) seems associated with lower AMI risk. Since the long-term AMI prognosis merits deeper investigation, we performed an observational study evaluating relationships between FXIIIA residual levels, cardiovascular risk-factors, and inherited genetic predispositions. FXIIIA V34L was genotyped in 333 AMI patients and a five-year follow-up was performed. FXIIIA levels assessed at day-zero (d0) and four days after AMI (d4), and conventional risk factors were analyzed, focusing on the development of major adverse cardiovascular events (MACE). FXIIIA assessed at d0 and d4 was also an independent MACE predictor in the long-term follow-up (FXIIIA_d0_, Odds Ratio (OR) = 3.02, 1.79–5.1, *p* = 0.013; FXIIIA_d4_, OR = 4.46, 2.33–8.55, *p* = 0.0001). FXIIIA_d4_ showed the strongest MACE association, suggesting that the FXIIIA protective role is maximized when high levels are maintained for longer time. Conversely, FXIIIA levels stratified by V34L predicted MACE at a lesser extent among L34-carriers (Hazard Risk (HR)_VV34_ = 3.89, 2.19–6.87, *p* = 0.000003; HR_L34-carriers_ = 2.78, 1.39–5.57, *p* = 0.0039), and V34L did not predict all MACE, only multiple-MACE occurrence (*p* = 0.0087). Finally, in survival analysis, heart failure and death differed significantly from stroke and recurrent ischemia (*p* = 0.0013), with FXIIIA levels appreciably lower in the former (*p* = 0.05). Overall, genetically-determined FXIIIA levels have a significant long-term prognostic role, suggesting that a pharmacogenetics approach might help to select those AMI patients at risk of poor prognosis in the need of dedicated treatments.

## 1. Introduction

After acute myocardial infarction (AMI), several processes aimed at repairing the damaged heart take place. Among these, the formation of a provisional 3D-fibrin scaffold is crucial for proper lesion healing, representing the ideal milieu for reparative cells recruitment and local secretome establishment [1]. The lack of activation of cardioprotective mechanisms is a key element to be investigated for the recognition of predictors for short- and long-term survival, and adverse event occurrence. In a short-term survival study, we recently demonstrated that after AMI, Factor XIIIA (FXIIIA) circulating levels are an independent predictor of major adverse cardiovascular events (MACE), in particular for heart failure (HF) and death [2]. On the contrary, the role of *F13A1* gene variants in AMI onset, recurrence, and in post-AMI outcomes is still controversial, including gender disparities [3,4,5,6,7,8].

FXIII zymogen circulates in plasma as a protransglutaminase complex of two enzymatic A-subunits, and two carrier B-subunits (FXIIIA2B2) that prevent the early and wasteful activation or degradation in blood of the active A-subunits [1]. In the classical model of coagulation, FXIIIA cross-links fibrin, supporting platelet (PLT) adhesion to damaged tissue and allowing the maintenance of an elastic architecture. Due to a wide category of FXIIIA substrates, most of them belonging to the extracellular matrix (ECM), in recent decades, FXIII has gained increasing attention in regenerative medicine, including processes related to lesion healing, angiogenesis, tissue repairing, and infections [1,9,10,11,12,13,14]. The FXIII molecule is now included among novel candidate biomarkers useful in prognostication of several complex diseases in which cardioprotection is a challenging goal [15]. Basically, inherited homozygous FXIII deficiency and defects in the *F13A1* gene result in bleeding complications and inefficient wound healing [9,10,16]. FXIIIA and other growth factors (such as those present in PLT) are particularly relevant during normal healing, and after AMI they support proper recovery of heart function, and contrast the ischemia-reperfusion injury [12,17,18,19,20]. In addition, the *F13A1* gene is characterized by a wide allelic heterogeneity, with several single nucleotide polymorphisms (SNP) of different functional and pharmacogenetic importance [16,21,22,23]. A common G–to–T polymorphism (rs5985) in the exon 2 of the *F13A1* gene causes a valine (V) to leucine (L) change at codon 34. This site is only three amino acids from the thrombin cleavage site (R37-G38), and the polymorphism significantly influences the activation rate by thrombin, changing the fibrin stabilization rate [24,25]. The Valine-to-Leucine (V34L) substitution could be particularly relevant during any ischemic event affecting the residual FXIIIA circulating levels, and in turn the quality of healing of the injured tissue, and the clinical outcome [2,3,26,27,28,29,30].

Although V34L is the most studied SNP and is considered the main functional locus among the several *F13A1* gene variants [31], its role in thrombosis and in clinical contexts is quite controversial [32,33], and might depend on the specific population and disease considered [34,35,36,37,38,39,40,41]. Accordingly, our group demonstrated that AMI patients had lower MACE occurrence and better one-year survival among L34-carriers compared to the VV34 homozygotes, also in combination with a second SNP (i.e., FXIIIB H95R) [3]. Moreover, we demonstrated that V34L could act as a protective inherited predisposition to ischemic diseases, as well as a risk factor predisposing to intracerebral hemorrhage [41]. Finally we showed that low circulating FXIIIA levels in the first days after AMI are associated with the worst prognosis in a short-term survey [2], in line with other reports focused on different pathological contexts [29,42,43].

We here report the extended five-year follow-up of AMI patients, focusing on the relationship between residual circulating levels of FXIIIA and conventional risk factors stratified by the FXIIIA V34L genotype, with the aim of recognizing novel molecular predictive biomarkers and therapeutic strategies useful for cardioprotection programs.

## 2. Results

### 2.1. Patient Characteristics

Baseline characteristics of enrolled patients stratified by MACE occurrence are reported in Table 1. The patient age range was 69.2 ± 12.7 years, 29.4% were female, 240 (72.1%) had ST-elevation myocardial infarction (STEMI), and 115 patients (34.53%) developed MACE within the five-year follow-up period. MACE+ patients were significantly older and had mean left ventricular ejection fraction (LVEF) significantly lower than MACE− patients. Moreover, they had a significantly lower rate of family history of cardiovascular events as well as smoking habits, while they showed higher arterial hypertension, diabetes, and previous myocardial infarction rates. Fibrinogen and C-reactive protein (CRP) circulating levels were both significantly higher in MACE+, as were Troponin I (TnI) and, to a lower extent, creatine kinase-MB (CK-MB) peak values (Table 1). Additional clinical details stratified by FXIIIA genotypes are shown in Supplementary Table S1. Noteworthy is the observation that MACE+ showed significantly lower total cholesterol and Low-density lipoprotein LDL than the MACE− counterpart (Supplementary Table S1).

### 2.2. FXIIIA Levels, Genotypes, and MACE

Globally, the FXIIIA L34 variant was present in 126 patients (109 VL34-heterozygotes and 17 LL34-homozygotes), yielding a carrier frequency of 37.8% (32.7% VL34 and 5.1% LL34) and a L34-allele frequency of 0.215. The remaining 207 patients were homozygous for the common allele (VV34, 62.2%). Neither the genotype distribution nor the number of L34-carriers versus VV34 homozygote comparisons yielded significant differences between MACE+ and MACE− patients (Table 2). Among MACE+ patients, 47 were L34-carriers (40.9%) and the remaining 68 were VV34 homozygotes (59.1%). Likewise, among MACE−, 79 were L34-carriers (36.2%) and the remaining 139 were VV34 homozygotes (63.8%). Interestingly, among L34-carriers (*n* = 126), females clustered in the MACE+ subgroup if compared with males (♀/♂ ratio: MACE+, 20/27; MACE−, 20/59) accounting for a more than twofold increased risk of experiencing any MACE for those females carrying the L34-allele (OR = 2.18, 1.01–4.7, *p* = 0.05).

FXIIIA levels at day-four were significantly lowered compared to day-zero in the entire AMI group regardless of the presence of MACE, as well as in the L34- or VV34-subgroups (Table 2). It is noteworthy that subanalysis of FXIIIA levels within the different FXIIIA genotypes always ascribed to L34-carriers the lowest values both at day-zero and at day-four. Of note, in the MACE+ subgroup this difference did not reach statistical significance, probably because MACE+ patients presented at day-zero with the lowest FXIIIA levels, regardless of the FXIIIA genotypes.

**Figure 1:**
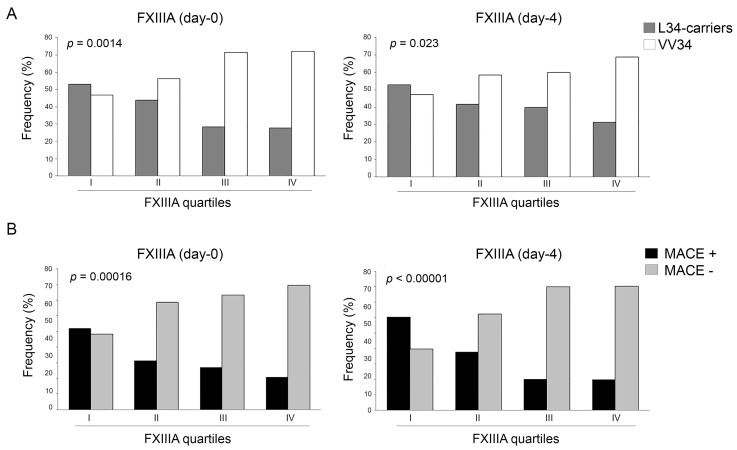
(**A**) Distribution of patients carrying FXIIIA VV34 genotype or L34-allele stratified by FXIIIA quartiles (I-IV) assessed at baseline (d0; left panel) and four days (d4; right panel) after AMI. Patients having FXIIIA levels in the first quartile had a non-significant slight overrepresentation of L34-carriers both at d0 and at d4. Their frequency significantly decreased from the first to the fourth quartile, the opposite of the frequency for the VV34 genotype (*p* = 0.0014 and *p* = 0.023 at d0 and d4, respectively); (**B**) Distribution of patients with and without major adverse cardiovascular events (MACE+ and MACE−) stratified by FXIIIA quartiles (I-IV) assessed at baseline (d0; left panel) and four days (d4; right panel) after AMI. Patients having FXIIIA levels in the first quartile had a non-significant slight overrepresentation of MACE+ at d0, whilst at d4 they were significantly overrepresented (*p* = 0.0001). MACE+ frequency significantly decreased from the first to the fourth quartile, the opposite to that of MACE− (*p* = 0.00016 and *p* < 0.00001 at d0 and d4 respectively).

**Figure 2:**
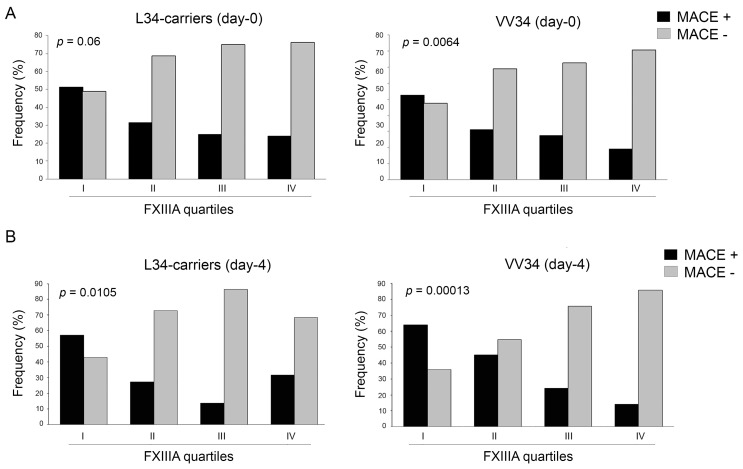
(**A**). Distribution of patients with and without major adverse cardiovascular events (MACE+ and MACE−) stratified by FXIIIA quartiles (I-IV) assessed at baseline (d0) among L34-carriers (left panel) and VV34-homozygotes (right panel). Patients having FXIIIA levels in the first quartile had a non-significant slight overrepresentation of MACE+ among L34-carriers or VV34-homozygotes. MACE+ frequency significantly decreased from the first to the fourth quartile, the opposite to that of MACE−, and this was more evident among VV34-homozygotes (*p* = 0.06 and *p* = 0.0064 among L34-carriers and VV34-homozygotes, respectively); (**B**) Distribution of patients with and without major adverse cardiovascular events (MACE+ and MACE−) stratified by FXIIIA quartiles (I-IV) assessed at day-four (d4) among L34-carriers (left panel) and VV34-homozygotes (right panel). Patients having FXIIIA levels in the first quartile had MACE+ overrepresented both among L34-carriers (*p* = 0.0036) and VV34-homozygotes (*p* = 0.0007). The fall in MACE+ occurrence was more evident among the VV34 genotype (*p* = 0.0105 and *p* = 0.00013 among L34-carriers and VV34-homozygotes, respectively).

By stratifying FXIIIA levels into quartiles (I-quartile = 25th percentile), those patients with FXIIIA within the I-quartile (FXIIIA_d0_ < 76.8% and FXIIIA_d4_ < 63.9%) showed a higher risk of developing any MACE (OR = 3.02, 1.79–5.10, *p* = 0.0138; and OR = 4.46, 2.33–8.55, *p* = 0.0001; at day-zero and day-four, respectively). As shown in Figure 1A, both at day-zero and day-four the number of L34-carriers significantly decreased as the levels of FXIIIA increased from the I to IV quartiles, whereas VV34 homozygotes showed the opposite trend. Similarly, with stronger significance, the rate of MACE occurrence was inversely related to FXIIIA quartiles both at day-zero and day-four (Figure 1B), and this was more evident among VV34 homozygotes (*p* = 0.0064) than in the L34-carriers (*p* = 0.06) (Figure 2A). However, the risk of experiencing any MACE having FXIIIA at day-zero within the I-quartile was comparable in both classes of genotypes (OR = 2.76, 1.27–5.99, *p* = 0.0109; and OR = 3.22, 1.55–6.68, *p* = 0.0018; for L34-carriers and VV34 homozygotes, respectively). Interestingly, the same risk-score calculation computed at day-four strongly increased in both genotypes (OR = 4.27, 1.65–10.99, *p* = 0.0036; and OR = 4.95, 1.96–12.47, *p* = 0.0007; for L34-carriers and VV34 homozygotes, respectively), as supported by the trends shown in Figure 2B, suggesting once more that FXIIIA reaches its full biomarker predictive role at day-four.

### 2.3. Survival Analysis and Predictive Model

**Figure 3:**
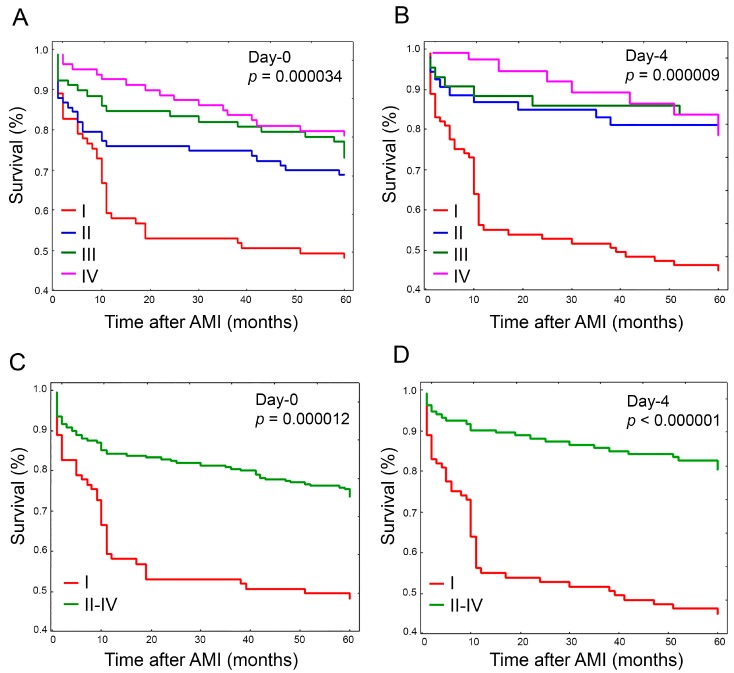
Plots of Kaplan-Meier survival analysis estimated after AMI according to FXIIIA quartiles assessed at baseline (day-zero) and four days after AMI (day-four). (**A**) Survival curve comparison according to FXIIIA quartiles I-IV measured at day-zero. At five-year follow-up, survival rates among the different quartiles were significantly different (*p* = 0.000034); (**B**) Survival curve comparison according to FXIIIA quartiles I-IV measured at day-four. At five-year follow-up, survival rates among the different quartiles were significantly different (*p* = 0.000009); (**C**) Survival curve comparison according to FXIIIA quartile I versus quartiles II-IV computed together at day-zero. At five-year follow-up, survival in the I quartile significantly differed from that of the II-IV quartiles (*p* = 0.000012); (**D**) Survival curve comparison according to FXIIIA quartile I versus quartiles II-IV computed together at day-four. At five-year follow-up, survival in the I quartile significantly differed from that of the II-IV quartiles (*p* < 0.000001).

**Figure 4:**
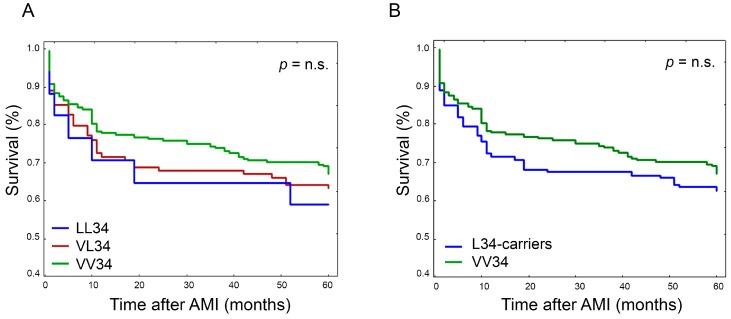
Plots of Kaplan-Meier survival analysis estimated after AMI according to FXIIIA V34L genotypes. (**A**) Survival curve comparison between LL34-homozygotes (LL34), VL34-heterozygotes (VL34), and VV34-homozygotes (VV34). Survival rate was not significantly different between the different genotypes; (**B**) Survival curve comparison between VL34-heterozygotes and LL34-homozygotes taken together (L34-carriers), and VV34-homozygotes (VV34). Survival rate was not significantly different between the two different groups of genotypes.

Survival analyses were conducted according to FXIIIA percentiles and different genotypes. Survival curves according to FXIIIA quartiles are shown in Figure 3A,B (day-zero and day-four, respectively). As expected, the risk associated with the development of any MACE in the five-year follow-up period was inversely related to FXIIIA levels, and at day-zero a clear stepwise trend was particularly evident (*p* = 0.000034) (Figure 3A). Conversely, at day-four, those patients with FXIIIA levels above the II quartile showed comparable survival rates that were higher than that of the I-quartile, in turn improving the significance level (*p* = 0.000009) (Figure 3B). This suggested we should combine the II-III-IV FXIIIA quartiles and compare them with the I-quartile, as shown in Figure 3C,D, respectively for day-zero and day-four. Accordingly, the overall risk (I-quartiles versus II-IV) was HR = 2.39, 1.62–3.54, *p* = 0.000012; and HR = 3.7, 2.28–5.96, *p* < 0.000001; at day-zero and day-four, respectively, suggesting again that FXIIIA reaches its full biomarker predictive role at day-four. Conversely, survival analysis performed stratifying patients by FXIIIA genotypes did not show any significant association of MACE with a particular genotype, either by considering each specific genotype or combining together L34-carriers (Figure 4A,B). Interestingly, low FXIIIA levels stratified according to the presence or absence of the polymorphic allele (i.e., VL + LL34 versus VV34) predicted MACE occurrence differently among the two groups, particularly at day-four (HR_VV34_ = 3.89, 2.19–6.87, *p* = 0.000003; HR_L34-carriers_ = 2.78, 1.39–5.57, *p* = 0.0039), and to a lesser extent at day-zero (HR_VV34_ = 2.32, 1.38–3.89, *p* = 0.0014; HR_L34-carriers_ = 2.14, 1.2–3.8, *p* = 0.0098). Although this difference was not statistically significant (*p* = 0.75), it confirmed that FXIIIA reaches its full biomarker predictive role at day-four.

**Figure 5:**
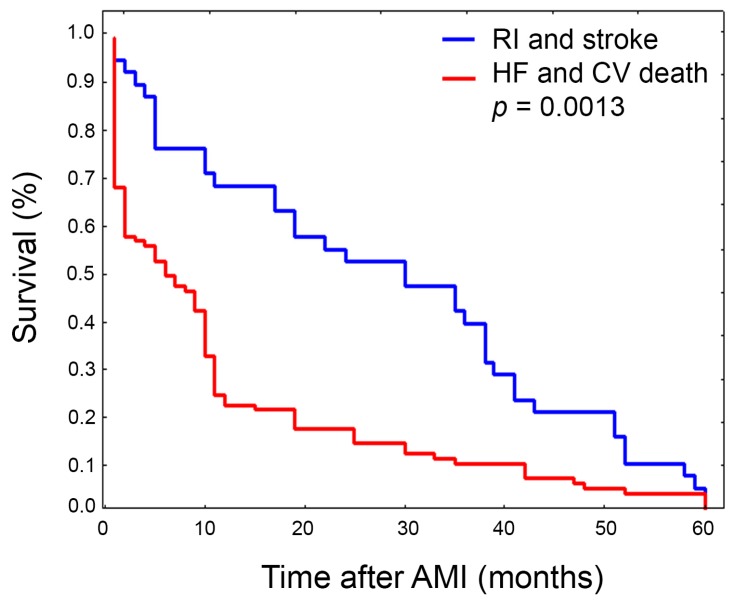
Plots of Kaplan-Meier survival analysis estimated after AMI according to different kinds of MACE at five-year follow-up. Cardiovascular (CV) death and heart failure (HF) combined together (red line), stroke and recurrent non-fatal ischemic events (RI) combined together (blue line). Survival significantly differed between subgroups (*p* = 0.0013).

Overall, during the five-year follow-up period there were a total of 139 MACE in 115 patients. The cardiovascular (CV) mortality and HF occurrence rates were 15.0% and 15.3%, respectively, whilst recurrent non-fatal ischemic events (RI) and stroke were less frequent, globally accounting for 11.4%. The majority of CV deaths and HFs occurred during the first year follow-up (75.2%), while RI and stroke showed a consistent trend over the entire follow-up period. In our previous work we suggested that presenting with non-optimal FXIIIA levels during the early phase of AMI could increase the risk of severe complications, mainly involving the integrity of the myocardial wall with respect to novel occurrence of nonfatal ischemic events. In order to verify this hypothesis, we stratified patients according to the different MACE and found that CV death and HF survival curves perfectly overlapped, as did those of stroke and RI. Subsequently, we combined CV death with HF, and stroke with RI (Figure 5), showing significantly different survival rates (*p* = 0.0013). In addition, although not reaching strong statistical significance mainly due to the low number of computable cases, patients experiencing HF or CV death had lower residual FXIIIA levels (*p* = 0.05). On the contrary, the frequency of L34-carriers was equally distributed among the two MACE subgroups (RI and stroke 43.0%, CV death and HF 42.05%). Of note, 24 patients had two or more MACE. Fourteen of them were L34-carriers (11.11% of L34-carriers) and only 10 cases were VV34 homozygotes (4.8% of VV34 homozygotes) yielding an OR = 2.46, 1.06–5.73, *p* = 0.047.

Finally, in the logistic regression model (univariate), those variables significantly associated to the endpoints were incorporated in multivariate analysis (Table 3) for both composite MACE (all MACE) and for MACE recurrence (multiple MACE). Of note, FXIIIA levels both at day-zero and at day-four resulted in independent predictors of MACE, and interestingly, levels within the 25th percentile (I-quartile) were significant predictors of composite-MACE (all MACE) in the multivariate analysis.

## 3. Discussion

Heart damage caused by AMI was considered an irreversible condition followed by scarring, affecting heart performance and survival. Timely reperfusion reduces acute mortality, but does not counteract left ventricular remodeling (LVR) or HF, and the long-term mortality or hospitalization rate remains high among AMI survivors [44,45,46]. We need to develop new treatments to limit LVR and HF, and discover novel candidate targets and early predictive biomarkers. Recently, the role of circulating transglutaminases FXIIIA in maintaining myocardium structural integrity created wide expectations. FXIIIA increases Vascular Endothelial Growth Factor (VEGF) expression, promoting in turn local angiogenesis and collagen synthesis, and also mechanically limiting infarct expansion [2,3,13,47,48]. In addition to VEGF, Insulin-like growth factor (IGF), and many other growth factors, platelets (PLT) contain high amounts of FXIIIA easily shuttled to the injury area, where fibrin entraps PLT and leukocytes at the heart lesion. PLT-rich plasma has been indeed suggested as an adjuvant treatment to save the myocardium wall [18,19,20]. This process promotes local secretome homeostasis, protects against Reactive Oxygen Species (ROS) generation, and stabilizes mitochondria during the ischemia-reperfusion injury, providing a favorable timely tuned niche only when optimal FXIIIA levels are maintained at the injured site. The key role of FXIIIA in maintaining heart integrity has been definitively demonstrated in *F13A1* gene knock-out mice that died due to heart rupture within five days of induced experimental AMI [12]. Contextually, we reported low residual circulating FXIIIA levels during AMI [3] in those patients who died earlier or developed severe HF [2]. Accordingly, the FXIIIA molecule must have a remarkable role also in LVR establishment after AMI. FXIIIA cross-links fibrin polymers in an elastic 3D-scaffold useful for recruitment, attachment, and differentiation of resident and circulating stem cells, neovascularization, cardiomyocyte survival, and decreased fibrosis [1].

The major finding of our research is that FXIIIA circulating levels are also independent predictors of MACE in the long-term after AMI, demonstrating that low FXIIIA levels are also predictors of poor outcomes after five-year follow-up. Interestingly, early assessment of FXIIIA also results in an independent predictor of MACE in the composite end-point of the multivariate analysis when considering values within the lowest percentile. Measurements at day-four very often increased the predictive power of FXIIIA, and this is strongly in line with the fact that some patients might keep normal or borderline levels of FXIIIA at day-zero in spite of successive MACE development. Of note, patients that do not show FXIIIA consumption in the earliest AMI phase have either a good chance of a good prognosis, or we speculated they could experience different kinds of MACE. Of particular interest are the data obtained by comparing CV death and HF combined together versus stroke and recurrent non-fatal ischemic events. They had significantly different trend rates, with the first two mainly occurring during the first months of follow-up, and the latter characterized by a more regular incidence during the entire follow-up period. In addition, the lower FXIIIA levels found among patients experiencing HF or CV death strongly matches with previous hypotheses that patients with excessive FXIIIA consumption during ongoing AMI died earlier or developed HF [1,2,3,12,13,47,48]. Accordingly, in our study we observed that presenting with non-optimal FXIIIA levels during the early phase of AMI considerably increased the risk of severe complications, mainly involving the integrity of the heart wall, and in particular LVR (unpublished data).

To analyze the combined effects of FXIIIA levels and genotype, in the present study we found interesting and mutually correlated findings. The inverse relation between L34-allele and FXIIIA levels among the different FXIIIA quartiles, together with the inverse relation between MACE occurrence and FXIIIA levels among the different FXIIIA quartiles, might suggest the presence of joint effects in terms of decreased FXIIIA levels as a consequence of L34-allele presence. The observation that after genotype stratification MACE occurrence is similarly related to FXIIIA levels in both genotypes, and that the association increased at day-four regardless of the genotype, suggests that additional factors are involved in the predisposition to MACE development via FXIIIA lowering. Accordingly, no significant associations were found stratifying MACE development by the V34L genotype, even though there is a slight trend of lower survival ascribed to L34-carriers (more evident among LL34-homozygotes). Moreover, although FXIIIA levels were lower among L34-carriers than in VV34 homozygotes, within the MACE+ subgroup this difference did not reach statistical significance, probably because MACE+ patients presented with low FXIIIA levels regardless of FXIIIA genotypes. This was particularly evident at day-four, where MACE+ VV34 homozygotes showed lower FXIIIA levels than MACE− L34-carriers, suggesting again that additional factors (together with L34-allele) might cause MACE occurrence via FXIIIA lowering.

Interestingly, in our population MACE+ patients presented with significantly lower total cholesterol and LDL levels than the MACE− counterpart. This data was also confirmed in the two different FXIIIA genotypes, though among L34-carriers the difference was not statistically significant. This implies that patients with high cholesterol and LDL levels experienced less MACE at the follow-up. This paradoxical result might be explained by considering the recent large metanalysis by Navarese and colleagues, who analyzed 34 randomized clinical trials that included 270,288 participants [49]. The authors reported a greater reduction in the risk of all cause cardiovascular mortality after cholesterol-lowering treatment in AMI patients with higher baseline LDL levels, and that a larger reduction in MACE was observed among those with higher baseline LDL levels and higher magnitude of LDL lowering [49].

Nowadays, the number of AMI survivors has dramatically increased thanks to prompt coronary reperfusion. On the other hand, such patients are considered orphans of dedicated therapies in the long term, and often develop HF and LVR. Accordingly, to translate into the clinical setting novel cardioprotective strategies, and also to discover novel therapeutic targets and pathways, we below summarize the key findings obtained in this extended five-year follow-up. Firstly, stratifying MACE events by FXIIIA quartiles, we found that survival within the lowest quartile was significantly lower than survival in the remaining quartiles, and a step-wise trend characterized the first year. Secondly, the presence of the several MACE events at day-zero among patients within the highest FXIIIA quartiles, and their contextual reduction at day-four, support the idea that only those patients who maintain high FXIIIA levels may benefit from its protective role that takes action mainly when high levels are sustained for longer.

This study corroborates the existence of an early specific time-window in which to operate to maintain optimal FXIIIA levels and sustain heart-repairing. At the same time, our approach will avoid the potential risk of indiscriminate FXIIIA treatment by means of monitoring FXIIIA dynamics early in the acute phase. This strategy together with a pharmacogenetics approach could be useful to recognize those patients really needing such adjuvant treatment [1,2,3,12,13,17,47,48].

## 4. Materials and Methods

### 4.1. Patients

The study included 333 patients admitted to the Coronary Care Unit of the University-Hospital of Ferrara with a diagnosis of AMI already involved in a previous study, aimed at assessing the dynamics of FXIIIA levels during the acute infarction phase [2]. In detail, the present study further genotyped 333 (95.1%) of the original 350 patients for FXIIIA V34L polymorphism and included an extended follow-up of five years and additional MACE evaluations.

AMI was defined according to the Joint ESC/ACCF/AHA/WHF Task Force for the Universal Definition of Myocardial Infarction [50], as a rise or fall (or both) of cardiac biomarkers (cardiac troponin I or T, CK-MB fraction of creatine kinase and CK-MB, measured by mass assay) with at least one of the following: symptoms of ischemia, new or presumed-new significant ST-segment-T wave changes or new left bundle branch block, development of pathological Q-waves in the electrocardiogram (ECG), imaging evidence of new loss of viable myocardium or new regional wall motion abnormality, or identification of an intracoronary thrombus by angiography. Patients with ST-elevation myocardial infarction (STEMI) received primary percutaneous coronary intervention (PCI) within 120 min of first medical contact, in both the case of symptoms ≤12 h in duration, and symptoms lasting 12 to 24 h if pain was present at the time of admission. Patients with non-STEMI (NSTEMI) underwent coronary angiography within 2 to 72 h of hospital admission, according to the European Society of Cardiology (ESC) recommendations for invasive evaluation and revascularization of NSTEMI coronary-syndromes. All patients received standard medical therapy according to the ESC guidelines for the treatment of AMI unless contraindicated, including aspirin, clopidogrel, glycoprotein IIb/IIIa inhibitors (tirofiban or abiciximab), unfractioned or low-molecular-weight heparin, betablockers, statins, and ACE inhibitors or angiotensin receptor blockers (or both) [44]. The baseline demographic, clinical, echocardiographic, and angiographic test results were collected from all patients. The study was carried out according to the Code of Ethics of the World Medical Association (Declaration of Helsinki) and was approved by the local ethics committee of the University Hospital of Ferrara (project identification code 070592, approved 28 June 2007 and 27 November 2008); all patients gave written informed consent to enter the study.

### 4.2. Blood Samples and FXIIIA Level Measurements

Peripheral venous blood samples were drawn at admission (d0) and every 24 h for the additional five days (d1–d5) from the confirmed AMI event to peak the highest FXIIIA consumption level. We found day-four (d4) as the most informative point and used it for all the statistical analyses. FXIIIA antigen circulating levels were assessed by Latex Reagent (HemosIL Factor XIII Antigen) on the automated Coagulation Analyzer (ACL Futura Plus) according to the manufacturer’s instructions (Instrumentation Laboratory, Milan, Italy), as previously reported [2]. According to our internal protocols, confirmation of FXIIIA levels was carried out by re-assaying random samples with different immunological procedures, as previously described [51,52]. There were no discrepancies between FXIIIA levels assessed in duplicate.

### 4.3. Genotype Analysis

Blood was collected at admission to avoid loss of cases (d0). Genomic DNA was obtained from peripheral blood, and genotyping for the FXIIIA V34L (rs5985) SNP was performed by PCR-amplification followed by the Pyrosequencing® technique (Pyromark ID System, Biotage AB Uppsala, Sweden). 

The primers used were respectively, Forward (Fw) 5′-AATGCAGCGGAAGATGACC-3′, Reverse (Rv) 5′-Biotynilated-GCTCATACCTTGCAGGTTGAC-3′, and Sequencing (Sq) primer 5′-CACAGTGGAGCTTCAG-3′. Using the Fw and Rv primers the product amplicon length was 77 bp. PCR conditions were as follows: Initial 5 min at 94 °C; followed by 40 cycles of 94 °C for 30 s, 52 °C for 22 s, and 72 °C for 15 s; and a 5 min final extension step at 72 °C. All PCR cycles were performed in a Sure Cycler 8800 (Agilent Technologies, California, CA, USA) using Taq DNA Polymerase (La Roche Ltd., Switzerland, CH). The sequencing analysis was performed using PyroMark ID (Qiagen, Maryland, MD, USA) according to the instrumental instructions, analyzing the following target sequence: GGC**G**/**T**TGGTGCCCCGGGGC (in bold, nucleotide variation; G = V34, T = L34). Confirmation of genotypes was carried out by re-genotyping random samples by DNA restriction, according to our internal protocols as previously described [53,54]. There were no discrepancies between genotypes determined in duplicate.

### 4.4. Follow-Up and Description of Endpoints

The primary endpoint was a composite of major adverse cardiovascular events (MACE) consisting of cardiovascular (CV) death, recurrent episodes of myocardial ischemia (RI, including non-fatal myocardial re-infarction or unstable angina), heart failure (HF), and stroke at the five-year follow-up. The CV events were defined according to the European Society of Cardiology (ESC) guidelines and the Standardized Definitions for Cardiovascular and Stroke End Point Events in Clinical Trials of the Clinical Data Interchange Standards Consortium (CDISC) [44,45,46,55].

### 4.5. Statistics

Continuous variables were expressed as the mean ± standard deviation (SD), and differences between groups were evaluated by Student’s T-test or the Mann–Whitney U-test. Categorical variables were expressed in percent frequency, and compared using the Chi-square test or Fisher’s exact test. One-way analysis of variance was used to compare continuous variables and genotype groups. Patients of the three genotype groups were computed in two categories: VV34 (non-carriers) versus VL34 and LL34 taken together (L34-carriers). Survival curves (MACE-free survival) were constructed by Kaplan-Meier analysis, and comparison of survival curves was performed with the log-rank test computing the different MACE together unless otherwise specified. MACE were retrospectively analyzed as a single variable, or combined by means of logistic regression analyses. Probability was considered significant at a level of *p* ≤ 0.05. Analysis was performed using MedCalc version 11.2.1.0 statistics software.

## Figures and Tables

**Table 1:** Baseline characteristics of patients with (MACE+) and without (MACE−) major adverse cardiovascular events.

Clinical and Demographic Characteristics	Whole Cohort (*n* = 333)	MACE+ (*n* = 115; 34.5%)	MACE− (*n* = 218; 65.5%)	*p*
Age, years (mean ± SD)	69.2 ± 12.7	73.8 ± 11.34	65.01 ± 12.22	**<0.0001**
Male/female (*n*) (female %)	235/98 (29.4)	75/40 (34.8)	160/58 (26.6)	n.s.
STEMI/NSTEMI (% *stemi*)	240/93 (72.1)	73/42 (63.5)	167/51 (76.6)	**0.0145**
LVEF (%, mean ± SD)	44.8 ± 11.0	40.9 ± 12.1	46.9 ± 10.0	**<0.0001**
Family History (%)	33.9	23.9	39.2	**0.0065**
Hypertension (%)	66.4	76.1	61.2	**0.0092**
Dyslipidaemia (%)	36.0	34.5	36.8	n.s.
Diabetes (%)	23.2	37.2	15.8	**0.0001**
Smoking Habit (%)	54.7	46.9	58.8	**0.046**
Previous MI (%)	27.5	48.7	16.3	**0.0001**
Fibrinogen (mg/dL)	313.4 ± 107.6	355.1 ± 122.1	296.8 ± 91.4	**<0.0001**
CRP (mg/dL)	2.6 ± 4.3	3.62 ± 5.16	2.05 ± 3.7	**0.002**
TnI peak value (ng/mL, mean ± SD)	4.92 ± 6.3	5.47 ± 7.3	3.9 ± 5.0	**0.025**
CK-MB peak value (ng/mL, mean ± SD)	137.9 ± 159	141.4 ± 205	136.1 ± 163	n.s.

LVEF indicates left ventricular ejection fraction; CRP indicates C-reactive protein; TnI and CK-MB indicate troponin I and creatine kinase-MB, respectively; n.s. indicates not significant.

**Table 2:** FXIIIA levels assessed at admission (d0) and four days after AMI (d4) in the whole group, and in the VV34- and L34-carriers stratified by presence (MACE+) or absence (MACE−) of major adverse cardiovascular events.

FXIIIA Genotype and Level	Whole Group (*n* = 333)			MACE+ (*n* = 115)			MACE− (*n* = 218)			*p* *
**FXIIIA V34L** *n* (%)	**VV34**	**VL34**	**LL34**	**VV34**	**VL34**	**LL34**	**VV34**	**VL34**	**LL34**	
	207 (62.2)	109 (32.7)	17 (5.1)	68 (59.1)	40 (34.8)	7 (6.1)	139 (63.8)	69 (31.7)	10 (4.6)	n.s.
	207 (62.2)	126 (37.8)		68 (59.1)	47 (40.9)		139 (63.8)	79 (36.2)		n.s.
**FXIIIA-d0** (%, mean ± SD)	99.4 ± 29.8			90.2 ± 29.4			103.8 ± 29.0			**<0.0001**
**FXIIIA-d4** (%, mean ± SD)	85.5 ± 29.8			74.5 ± 28.0			91.4 ± 28.4			**<0.0007**
***p***	**<0.0001**			**<0.0001**			**<0.0001**			
	**VV34**	**L34-carriers**	***p***	**VV34**	**L34-carriers**	***p***	**VV34**	**L34-carriers**		***p***
**FXIIIA-d0** (%, mean ± SD)	103.7 ± 28.6	92.4 ± 30.4	**0.00092**	93.5 ± 26.6	85.8 ± 32.6	n.s.	108.2 ± 28.3	96.1 ± 28.6		**0.0029**
**FXIIIA-d4** (%, mean ± SD)	90.1 ± 26.6	78.9 ± 26.7	**0.0029**	77.7 ± 26.5	69.8 ± 31.1	n.s.	96.8 ± 24.4	83.9 ± 22.6		**0.0022**
***p***	**<0.00001**	**0.00096**	-	**0.0034**	**0.037**	-	**0.0027**	**0.0085**		-

* *p* values refer to the comparison between MACE+ and MACE−; n.s. indicates not significant.

**Table 3:** Logistic regression for composite–MACE and MACE–recurrence.

Clinical and Demographic Characteristics	Composite–MACE (All MACE)		MACE–Recurrence (Multiple MACE) *	
	*p* (Univariate)	*p* (Multivariate)	*p* (Univariate)	*p* (Multivariate)
Age	**<0.0001**	**0.040**	n.s.	--
Sex (male)	**0.020**	n.s.	--	--
FXIIIA_d0_	**0.0002**	n.s.	n.s.	--
FXIIIA_d4_	**0.0012**	n.s.	n.s.	--
FXIIIA_d0_ < 76.8%	**0.00006**	**0.004**	n.s.	--
FXIIIA_d4_ < 63.9%	**0.000001**	**0.0001**	n.s.	--
FXIIIA V34L	n.s.	--	**0.0195**	**0.0087**
Total cholesterol	**<0.0001**	n.s.	n.s.	--
LDL cholesterol	**0.0019**	n.s.	n.s.	--
HDL cholesterol	n.s.	--	n.s.	--
Triglycerides	n.s.	--	n.s.	--
Serum creatinine—baseline	**<0.0001**	n.s.	n.s.	--
Serum creatinine—peak value	**<0.0001**	n.s.	n.s.	--
Fibrinogen	**<0.0001**	n.s.	n.s.	--
Previous AMI	**<0.0001**	**0.0057**	n.s.	--
Diabetes	**<0.0001**	n.s.	n.s.	--
TnI *day-1*	**0.0382**	n.s.	n.s.	--
CK-MB *day-3*	**0.0148**	n.s.	**0.0438**	**0.0441**
CRP	**0.0046**	n.s.	n.s.	--
Family history	**0.0060**	n.s.	n.s.	--
Smoking habit	**0.0404**	n.s.	n.s.	--
Hypertension	n.s.	--	n.s.	--

CRP indicates C-reactive protein; TnI and CK-MB indicate troponin I and creatine kinase-MB, respectively; FXIIIA_d0_ < 76.8% and FXIIIA_d4_ < 63.9% indicate the I quartile of FXIIIA at day-zero and day-four, respectively; n.s. indicates not significant. * indicates two or more MACE.

## Supplementary Materials

Supplementary materials can be found at http://www.mdpi.com/1422-0067/19/9/2766/s1.
