# Supplementary material for: F13A1 Gene Variant (V34L) and Residual Circulating FXIIIA Levels Predict Short- and Long-Term Mortality in Acute Myocardial Infarction after Coronary Angioplasty
Source: Int J Mol Sci. 2018 Sep 14;19(9):2766. doi: 10.3390/ijms19092766 (PMC6165350; doi:10.3390/ijms19092766)
Supplement: Supplementary file 1 [file ijms-19-02766-s001.pdf]

International Journal of Molecular Sciences

MDPI

1 Supplementary Table 1. Laboratory and clinical features of patients according to presence (MACE+) or absence (MACE-) of major adverse cardiovascular events.

Laboratory and angiographic data	Whole population (n=333)			L34-carriers (n=126, 37.8%)			VV34 homozygotes (n=207, 62.2%)		
	**MACE +**	**MACE -**	***P***	**MACE +**	**MACE -**	***P***	**MACE +**	**MACE -**	***P***
**Creatinine basal value** (mg/dl, mean ±SD)	1.46 ± 1.4	0.96 ± 0.4	**<0.0001**	1.4 ± 0.9	0.9 ± 0.2	**0.0001**	1.5±1.7	1.0 ± 0.4	**0.0011**
**Creatinine peak value** (mg/dl, mean ± SD)	1.84 ± 1.7	1.1 ± 1.0	**<0.0001**	1.9 ± 1.6	1.0 ± 0.3	**<0.0001**	1.8±1.9	1.1 ± 0.8	**0.0018**
**Total Cholesterol** (mg/dl, mean ± SD)	169.0 ± 41.4	195.6 ± 42.6	**<0.0001**	171.5 ± 43.6	185.7 ± 42.6	n.s	168.6±41.2	201.2 ± 47.8	**<0.0001**
**HDL** (mg/dl, mean ± SD)	44.7 ±14.6	46.5 ± 12.5	n.s.	45.0 ± 12.9	44.9 ± 11.3	n.s	44.5 ± 15.7	45.9 ± 12.0	n.s
**LDL** (mg/dl, mean ± SD)	99.4 ± 34.7	122.8 ± 42.3	**<0.0001**	102.3 ± 38.4	115.2 ± 38.3	n.s	98.7 ± 33.8	127.1 ± 48.0	**0.0002**
**Triglycerides** (mg/dl, mean ± SD)	124.5 ± 61.6	136.1 ± 69.9	n.s.	121.2 ± 56.7	127.6 ± 59.4	n.s	126.8 ± 64.7	141.3 ± 75.9	n.s
**Glycated hemoglobin** (%, mean ± SD)	6.6 ± 1.4	6.3 ± 1.2	**0.014**	6.6 ± 1.2	6.2 ± 1.0	n.s	6.7±1.5	6.3±1.2	n.s
**Uric acid** (mg/dl, mean ± SD)	6.2 ± 1.9	5.91 ± 1.74	n.s	6.1 ± 1.9	5.6 ± 1.7	n.s	6.2±1.9	5.9±1.6	n.s
**CAD extent**									
**Single-vessel disease**	30.4	42.7	**0.03**	32	47	**0.0498**	29	40	n.s
**Double-vessel disease**	31.3	29.4	n.s	27	28	n.s	34	30	n.s
**Triple-vessel disease**	38.3	28.4	n.s	41	25	n.s	37	30	n.s
**N ° of stent**									
**0**	20.0	25.8	n.s	11	18	n.s	26	21	n.s
**1**	42.6	45.0	n.s	47	51	n.s	40	50	n.s
**2**	23.5	21.5	n.s	26	23	n.s	21	21	n.s
**3**	6.1	3.6	**0.0001**	5	5	n.s	7	3	n.s
**4 or more**	7.8	4.0	n.s	10	3	n.s	6	5	n.s
**Type of stent**									
**BMS**	45.2	33.9	**0.05**	55	36	**0.0333**	39	33	n.s
**DES**	36.5	44.9	n.s	37	46	n.s	37	45	n.s
**POBA**	17.4	20.2	n.s	8	18	n.s	24	22	n.s

2 CAD = coronary artery disease; BMS = bare metal stent; DES = drug eluting stent; POBA = percutaneous only balloon angioplasty; n.s., indicates not significant.
